# Knowledge, attitudes, and practices of medical students at a Chinese university toward virtual simulation experimental training: a cross-sectional survey of self-perceived experience

**DOI:** 10.1186/s12909-026-08803-w

**Published:** 2026-02-12

**Authors:** Qing Xia, Juanjuan Bu, Hang Zuo, Jie Wang, Yuesong Hou, Jinian Wang, Xiaoli Wu

**Affiliations:** 1 Education Division, The First Affiliated Hospital of Anhui Medical University, Hefei, Anhui 230022 China; 2 Virtual Simulation Teaching and Research Section, The First Affiliated Hospital of Anhui Medical University, Hefei, Anhui 230022 China; 3 Office of the CPC Fuyang Hospital of Traditional Chinese Medicine Committee, Fuyang, Anhui 236001 China; 4 Hospital Director’s Office, The First Affiliated Hospital of Anhui Medical University, Hefei, Anhui 230022 China

**Keywords:** Attitude, Knowledge, Practice, Medical students, Simulation

## Abstract

**Background:**

Virtual simulation has emerged as an innovative teaching approach in medical education, offering immersive and repeatable practice opportunities that can address the limitations of traditional skills training. This study aimed to assess the knowledge, attitudes, and practices (KAP) of Chinese medical students toward virtual simulation experimental training.

**Methods:**

A cross-sectional survey was conducted from August to September 2025 at Anhui Medical University, China. Data were collected using a structured questionnaire, which included demographic information and KAP-related assessments.

**Results:**

A total of 525 valid responses were obtained from 536 distributed questionnaires, yielding a valid response rate of 97.95%. Among the 525 valid respondents, 245 (46.7%) were male and 280 (53.3%) were female. By grade, 212 (40.4%) were in the junior year, 164 (31.2%) in the senior year, and 149 (28.4%) in the fifth year. Regarding majors, 245 (46.7%) studied Clinical Medicine, 59 (11.2%) Radiology, 139 (26.5%) Medical Imaging, and 82 (15.6%) Anesthesiology. The mean scores for knowledge, attitude, and practice were 12.11 ± 2.40 (possible range: 3–15), 15.78 ± 3.26 (possible range: 4–20), and 12.22 ± 2.60 (possible range: 4–16), respectively. Multivariate linear regression revealed that knowledge and attitude were significantly associated with practices (β = 0.186, 95% CI: [0.101, 0.271] and β = 0.593, 95% CI: [0.537, 0.649], both *P* < 0.001). Mediation analysis showed that the direct effect of knowledge on practice remained significant (β = 0.191, 95% CI: [0.114, 0.268], *P* < 0.001), while the indirect effect through attitude was also significant (β = 0.437, Bootstrap 95% CI: [0.348, 0.548]), accounting for 69.59% of the total effect.

**Conclusion:**

Chinese medical students generally demonstrate adequate knowledge and positive attitudes, yet their actual engagement in virtual simulation training remained limited. Enhancing awareness and training could further promote its application and integration into medical education curricula.

**Clinical trial number:**

Not applicable.

**Supplementary Information:**

The online version contains supplementary material available at 10.1186/s12909-026-08803-w.

## Background

Virtual simulation experimental training is a teaching approach that utilizes computer technology, three-dimensional modeling technology, and virtual reality technology to recreate real-life scenarios [1]. Virtual simulation teaching by simulating authentic medical scenarios, provides an innovative learning mode encompassing various phases, from basic medical studies to clinical practice skills training. It provides medical students with a safe, repeatable, and immersive environment to practice clinical skills, thereby compensating for the limitations of traditional lecture and demonstration based teaching [2]. In contrast to traditional lecture-based and demonstration-based methods, virtual simulation technology employed in clinical skills training can closely replicate real medical scenes, so that the students can repeatedly practice and refine their skill operations in a secure clinical environment [3], enhance the enthusiasm for independent learning [4] and reduce medical errors in clinical operations [5]. As medical education worldwide shifts toward digital and competency-based models, virtual simulation has become an important supplement to traditional training [2, 6].

Meanwhile, virtual simulation technology plays a crucial role in boosting learning outcomes. Especially in the realm of medical education, its impact is at a medium to high level [7]. In China, the government has actively promoted education informatization, leading to the establishment of national virtual simulation teaching centers and platforms. Many universities are gradually integrating virtual simulation teaching content into their curricula [8]. Following the COVID-19 pandemic, Chinese medical education has experienced an accelerated shift toward online, blended, and digital learning models. Restrictions on in-person teaching and clinical access during the pandemic further highlighted the need for flexible and scalable training approaches, making virtual simulation an increasingly important solution for maintaining teaching continuity and practical skills training. As a result, virtual simulation has become a prominent and rapidly developing topic in recent medical education reforms in China. Nevertheless, the application remains uneven, with limited resources, inconsistent implementation, and insufficient evaluation [9]. International research has primarily focused on the effectiveness of specific virtual simulation courses or technical aspects [10]. In contrast, there is still a paucity of studies assessing how medical students, as end-users, perceive and engage in virtual simulation instruction in China.

Knowledge, Attitude and Practice (KAP) theory is widely applied to explore how individuals’ cognition and perceptions influence their behaviors [11, 12]. Compared with technology-oriented frameworks such as the Technology Acceptance Model (TAM), which mainly focus on perceived usefulness and ease of use, the KAP framework provides a more comprehensive behavioral perspective by integrating cognitive understanding, attitudinal disposition, and actual behavioral engagement. This makes KAP particularly suitable for evaluating educational interventions that aim to promote not only technology acceptance but also learning participation and skill-related practice outcomes. In the context of virtual simulation training, KAP allows for a systematic assessment of how students’ knowledge acquisition and attitudinal responses translate into practical learning behaviors. However, there is a noticeable paucity of studies addressing the integrated analysis of knowledge, attitude and practice factors in research on virtual simulation teaching and learning. In a large amount of related literature, the emphasis lies primarily on specific courses or technological assessments. Few studies have simultaneously explored the knowledge, attitudes, and practices of medical students specifically in the context of virtual simulation teaching, whereas most existing work on virtual simulation in undergraduate medical education focuses on technological applications or outcomes rather than KAP dimensions [13]. Additionally, although KAP studies have been conducted in related areas of clinical education (e.g., simulation use among nursing educators), such work does not directly address medical students’ perspectives toward virtual simulation experiments [14]. Therefore, based on this framework, we hypothesized that medical students with higher knowledge and more positive attitudes toward virtual simulation would demonstrate stronger engagement and practices in training. This study employed a questionnaire survey to investigate Chinese medical students’ KAP toward virtual simulation experimental training instruction.

## Methods

### Study design and participants

This cross-sectional study was conducted from August to September 2025 at Anhui Medical University, Hefei City, Anhui Province, China. Ethical approval was obtained from the Ethics Committee of the First Affiliated Hospital of Anhui Medical University (Approval No. PJ 2025-05-84). All participants provided informed consent prior to completing the survey. Since students majoring in Clinical Medicine usually take basic medical courses during their freshman and sophomore years, and only begin to take Clinical Medicine-related courses in their junior year, the inclusion criteria were: 1) Students who voluntarily agreed to participate and could complete the questionnaire independently. The exclusion criteria were: 1) Students unable to complete the questionnaire due to absence or technical reasons.

### Questionnaire introduction and quality control

The questionnaire design was based on the best evidence summary of virtual simulation experimental training among medical education professionals [15–17]. The pilot test was carried out among medical students, with a total of 79 participants. The scale showed good reliability and validity, with a Cronbach’s alpha coefficient of 0.896. In addition, the questionnaire items were reviewed by a panel of medical education and clinical teaching experts to assess content relevance and clarity. Expert feedback was used to refine wording and ensure the appropriateness of item content prior to the main survey. The Cronbach’s alpha coefficient of the questionnaire in this study was tested to be 0.912 and the KMO value was 0.907, indicating that the questionnaire has good reliability and validity.

Following an extensive review of literature and in-depth expert discussions, we developed a questionnaire for collecting data on medical students’ knowledge, attitudes and practices concerning virtual simulation instruction. The questionnaire was structured into two distinct sections, encompassing a total of 18 questions. The first part was designed to collect demographic information about the participants, including their gender, grade, and major. The second part consisted of three sets of questions on knowledge, attitudes and practices regarding virtual simulation instruction, including five knowledge-based surveys (K1-K5), five attitude-based surveys (A1-A5), and five practice-based surveys (P1-P5). Points were assigned based on the number of options for each item. The knowledge, attitude and practice dimensions employed a scale with five response options, ranging from very positive (5 points) to very negative (1 point). For Question P1, a dichotomous item, a score of 1 was assigned to the response “Yes”, while a score of 0 was assigned to “No”. Multiple-select questions (K4, K5, A5, P5) were scored 0 points and were used solely for qualitative description. This scoring approach was determined through expert panel discussion and aligned with established practices in KAP research. In KAP studies, scoring methods are typically designed based on the nature of the items and study objectives, with knowledge items scored based on correctness to reflect respondents’ actual knowledge level [18]. Additionally, methodological guidelines for KAP surveys emphasize careful design and scoring procedures developed with expert input to ensure the validity and interpretability of results [19]. Scores equal to or exceeding 60% of the maximum possible score in each dimension were considered indicative of adequate knowledge, positive attitude, and proactive practice, respectively. Students meeting the inclusion criteria received the electronic questionnaire via WeChat groups. The survey utilized an online questionnaire from the “SoJump” (https://www.wjx.cn/) application within WeChat, accessible through a generated QR code. Participants logged in by scanning the QR code sent via WeChat and completed the questionnaire.

Research assistants received comprehensive training on the entire survey process, including how to explain the questionnaire to participants, distribute and collect responses, provide instructions for completion, and manage data export. During administration, they addressed participants’ questions to ensure full understanding of each item and the overall purpose of the survey. To maintain data quality, each IP address was limited to a single submission, all items were mandatory, and duplicate questions were avoided. After collection, investigators carefully reviewed questionnaires for completeness, internal consistency, and logical accuracy. Prior to statistical analysis, rigorous data cleaning was performed. Questionnaires were excluded if they were duplicated, contained implausible values (e.g., unrealistic ages or illogical response patterns such as identical selections throughout), were incomplete, or were completed in less than 10 s.

### Statistical analysis

Data analysis was conducted using SPSS 27.0 (IBM Corp., Armonk, N.Y., USA). Continuous variables were summarized as mean ± standard deviation (SD), while categorical variables were presented as number and percentage (n, %). Multivariate linear regression was employed to analyze the relationship between the knowledge, attitudes and practices. The dependent variables included knowledge, attitudes and practices, which were calculated by summing up the scores of the corresponding questions. The independent variables covered gender, major, and grade. In Model 1, the analysis centered on the knowledge aspect related to the independent variables; Model 2 delved into the attitudes towards the independent variables, incorporating the knowledge factor among the independent variables. Model 3 analyzed the practices associated with the independent variables, taking into account both the knowledge and attitude factors within the independent variables. Only students who responded “Yes” to item P1 (indicating participation in virtual simulation teaching) were included in the subsequent analysis of items P2 and P3, with non-participants excluded. Mediation effect analysis was performed using the PROCESS macro, with the Bootstrap method and a Bootstrap sample size of 5000. It was hypothesized that knowledge directly affects attitude and practice, while attitude directly affects practice. A two-sided P-value < 0.05 was considered indicative of statistical significance.

## Results

### Basic characteristics

A total of 536 questionnaires were distributed in this study and 525 valid questionnaires were reclaimed with a valid reclaim rate of 97.95%. A total of 11 invalid questionnaires were excluded. Specifically, 5 questionnaires with a response time of less than 10 s, 4 duplicate submissions, and 2 questionnaires with identical selections throughout were excluded. Among the participants, 245 (46.7%) were male and 280 (53.3%) were female. By grade, 212 (40.4%) were in the junior year, 164 (31.2%) in the senior year, and 149 (28.4%) in the fifth year. Regarding majors, 245 (46.7%) studied Clinical Medicine, 59 (11.2%) in Radiology, 139 (26.5%) in Medical Imaging, and 82 (15.6%) in Anesthesiology. The mean scores for knowledge, attitude, and practice were 12.11 ± 2.40, 15.78 ± 3.26, and 12.22 ± 2.60, respectively (Table 1).

**Table 1:** Basic characteristics

Variables	*N* (%)	Knowledge, mean ± SD	Attitude, mean ± SD	Practice, mean ± SD
		12.11 ± 2.40	15.78 ± 3.26	12.22 ± 2.60
Gender				
Male	245 (46.7%)	12.04 ± 2.54	15.73 ± 3.68	12.20 ± 2.98
Female	280 (53.3%)	12.17 ± 2.27	15.83 ± 2.84	12.24 ± 2.26
Grade				
Junior year	212 (40.4%)	12.42 ± 1.88	16.11 ± 3.01	12.70 ± 2.33
Senior year	164 (31.2%)	12.82 ± 1.94	15.85 ± 3.37	12.22 ± 2.74
Fifth year	149 (28.4%)	10.89 ± 3.00	15.26 ± 3.42	11.37 ± 2.61
Major				
Clinical Medicine	245 (46.7%)	11.68 ± 2.26	15.79 ± 3.23	11.76 ± 2.73
Radiology	59 (11.2%)	12.63 ± 1.79	15.32 ± 3.43	11.66 ± 2.84
Medical Imaging	139 (26.5%)	12.27 ± 2.70	15.72 ± 3.15	12.49 ± 2.45
Anesthesiology	82 (15.6%)	12.76 ± 2.43	16.22 ± 3.39	12.65 ± 2.48

### Knowledge of virtual simulation

Most respondents (81.70%) reported knowing about virtual simulation technology (K1: “Do you know about virtual simulation technology?”), and 84.20% were aware of medical virtual simulation training (K2: “Do you know about medical virtual simulation experimental training teaching?”). Additionally, 61.70% agreed that virtual simulation training is more advantageous than traditional skills training (K3) (Table 2). Regarding sources of information (K4: “What is the way you learned about virtual simulation?”), the majority cited course training (67.40%), followed by the Internet (49.10%), lectures or conferences (37.10%), and textbooks or journals (33.00%). Only 3.00% had never heard of virtual simulation. When asked about advantages (K5: “What do you think are the advantages of virtual simulation technology?”), students emphasized clinical simulation and immersion (75.20%), rich resources for independent learning (75.80%), human–computer interaction and timely feedback (72.20%), and safety for practical training (68.60%) (Table 3).

**Table 2:** Knowledge level of medical students about virtual simulation experimental training

*N*	Variables	Strongly disagree *n*(%)	Disagree *n*(%)	Not sure *n*(%)	Agree *n*(%)	Strongly Agree *n*(%)
K1	Do you know about virtual simulation technology?	11(2.1)	60(11.4)	25(4.8)	191(36.4)	238(45.3)
K2	Do you know about medical virtual simulation experimental training teaching?	10(1.9)	50(9.5)	23(4.4)	176(33.5)	266(50.7)
K3	Do you feel that virtual simulation experimental training instruction is more advantageous than traditional skills training?	9(1.7)	24(4.6)	168(32.0)	196(37.3)	128(24.4)

**Table 3:** Knowledge level of medical students about virtual simulation experimental training

*N*	Variables	Group	Frequency (*n*)	Proportion (%)
K4	What is the way you learned about virtual simulation?	Lectures, conferences	195	37.1
		Internet	258	49.1
		Textbooks, journals and publications	173	33.0
		Course training	354	67.4
		Recommended by others	58	11.0
		Never heard of it	16	3.0
K5	What do you think are the advantages of virtual simulation technology?	Clinical simulation, deeply immersive	395	75.2
		Human-computer interaction, timely feedback	379	72.2
		Rich resources, independent learning	398	75.8
		Safety guarantee, practical training	360	68.6

### Attitudes of virtual simulation

The majority (77.50%) expressed willingness to participate in virtual simulation training (A1: “Are you willing to participate in virtual simulation clinical skills training?”). Furthermore, 72.40% agreed that such training would stimulate learning enthusiasm and improve independent learning (A2). In terms of effectiveness (A3: “Do you think virtual simulation learning can achieve results comparable to real experiments and clinical practice?”), 63.10% responded positively, while 36.90% disagreed. Regarding dissemination, 43.80% agreed and 29.50% strongly agreed that they would recommend virtual simulation to others (A4) (Table 4). When asked about the most appropriate stage (A5: “At which stage do you think it is necessary to conduct virtual simulation for clinical learning?”), 76.40% students selected clinical specialty courses, 51.60% before specialty courses, and 40.80% the clinical internship stage (Table 5).

**Table 4:** Medical students’ attitudes toward virtual simulation experimental training

*N*	Variables	Strongly disagree *n*(%)	Disagree *n*(%)	Not sure *n*(%)	Agree *n*(%)	Strongly Agree *n*(%)
A1	Are you willing to participate in virtual simulation clinical skills training?	7(1.3)	9(1.7)	102(19.4)	227(43.2)	180(34.3)
A2	Do you have a positive attitude towards virtual simulation learning to stimulate learning enthusiasm and improve the efficiency of independent learning?	8(1.5)	14(2.7)	123(23.4)	218(41.5)	162(30.9)
A3	Do you have a positive attitude towards virtual simulation learning can achieve similar results with real experiments, diagnosis and treatment process?	14(2.7)	33(6.3)	147(28.0)	202(38.5)	129(24.6)
A4	Are you willing to recommend virtual simulation experimental training to others?	8(1.5)	11(2.1)	121(23.0)	230(43.8)	155(29.5)

**Table 5:** Medical students’ attitudes toward virtual simulation experimental training

*N*	Variables	Group	Frequency (*n*)	Proportion (%)
A5	At which stage do you think it is necessary to conduct virtual simulation for clinical learning?	Before learning clinical specialty courses	271	51.6
		When learning clinical specialty courses	401	76.4
		At the clinical internship stage	214	40.8
		Indifferent	21	4.0

### Practices of virtual simulation

More than half (56.40%) of respondents reported prior participation in a virtual simulation training course (P1: “Have you ever participated in a virtual simulation experimental training course?”). Among the 296 students who reported prior participation, 66.9% agreed or strongly agreed that their experimental or clinical skills had improved through participation (P2). Regarding substitution (P3: “Have you used virtual simulation courses to replace part of real experimental or clinical skills training?”), 56.1% indicated agreement or strong agreement. A large proportion (62.90%) hoped that virtual simulation training would become a compulsory undergraduate course (P4) (Table 6). When asked about learning pathways (P5: “In what way will you engage in learning through virtual simulation training?”), 89.10% reported participation through their school’s courses, 56.60% used public e-learning platforms, and 26.30% accessed courses offered by other schools (Table 7).

**Table 6:** Practices of medical students towards virtual simulation experimental training (P2 and P3 based on students who reported prior participation, *n* = 296)

*N*	Variables	Yes		No		
P1	Have you ever participated in a virtual simulation experimental training course?	296(56.4)		229(43.6)		

	**Variables**	**Strongly disagree** **n(%)**	**Disagree** **n(%)**	**Not sure** **n(%)**	**Agree** **n(%)**	**Strongly Agree** **n(%)**
P2	You have improved your experimental or clinical skills by participating in virtual simulation learning.	5(1.7)	15(5.1)	75(25.3)	127(42.9)	74(24.0)
P3	You attended virtual simulation courses to replace part of the real experimental or clinical skills training.	11(3.7)	31(10.5)	88(29.7)	100(33.8)	66(22.3)
P4	You hoped that the virtual simulation experimental training can be established as a compulsory course.	22(4.2)	24(4.6)	149(28.4)	204(38.9)	126(24.0)

**Table 7:** Practices of medical students towards virtual simulation experimental training

*N*	Variables	Group	Frequency (*n*)	Proportion (%)
P5	In what way will you learn through virtual simulation experimental practical training?	From their school’s virtual simulation course	468	89.1
		From the public virtual simulation e-learning platform	297	56.6
		From other school’s virtual simulation courses	138	26.3
		Other ways	5	1.0
		Indifferent	11	2.1

### Multivariate linear regression analysis

Multivariate linear regression analysis showed that knowledge was significantly higher among students majoring in Radiology (β = 0.842, 95% CI: [0.199–1.485], *P* = 0.010) and Anesthesiology (β = 0.825, 95% CI: [0.255–1.395], *P* = 0.005) compared with Clinical Medicine, while gender and grade had no statistical significance. Notably, fifth-year students demonstrated significantly lower knowledge scores compared with junior students (β=-1.474, 95% CI: [-1.948-0.999] *P* < 0.001). A partial explanation for this discrepancy lies in the limited on-campus participation of fifth-year students in structured virtual simulation training, given that these learners are primarily assigned to off-site clinical rotations and internships, thereby constraining their access to university-based simulation resources. Attitude was positively associated with knowledge (β = 0.703, 95% CI: [0.594, 0.812], *P* < 0.001) but differed by major, with Clinical Medicine students scoring higher than Radiology students (β=-1.140, 95% CI: [-1.957, -0.323], *P* = 0.006); gender and grade were not statistical significant. Practice scores were not significantly associated with gender, major, or grade. However, practice was significantly influenced by knowledge (β = 0.186, 95% CI: [0.101, 0.271], *P* < 0.001) and attitude (β = 0.593, 95% CI: [0.537, 0.649], *P* < 0.001) (Table 8).

**Table 8:** Multivariate linear regression analysis of knowledge, attitudes and practices

Variables	β	95%CI	*P*
**Model 1: knowledge**			
Gender			
Female	Ref	Ref	
Male	0.005	(-0.385,0.394)	0.981
Major			
Clinical Medicine	Ref	Ref	
Radiology	0.842	(0.199,1.485)	0.010
Medical Imaging	0.468	(-0.005,0.941)	0.052
Anesthesiology	0.825	(0.255,1.395)	0.005
Grade			
Junior	Ref	Ref	
Senior year	0.365	(-0.098,0.829)	0.122
Fifth year	-1.474	(-1.948,-0.999)	< 0.001
**Model 2: attitude**			
Gender			
Female	Ref	Ref	
Male	-0.078	(-0.569,0.414)	0.757
Major			
Clinical Medicine	Ref	Ref	
Radiology	-1.140	(-1.957,-0.323)	0.006
Medical imaging	-0.462	(-1.061,0.137)	0.130
Anesthesiology	-0.200	(-0.925,0.525)	0.588
Grade			
Junior	Ref	Ref	
Senior year	-0.584	(-1.171,0.002)	0.051
Fifth year	0.174	(-0.447,0.794)	0.582
Knowledge	0.703	(0.594,0.812)	< 0.001
**Model 3: practice**			
Gender			
Female	Ref	Ref	
Male	0.136	(-0.176,0.449)	0.392
Major			
Clinical Medicine	Ref	Ref	
Radiology	-0.076	(-0.646,0.494)	0.793
Medical Imaging	0.322	(-0.076,0.720)	0.112
Anesthesiology	0.012	(-0.445,0.469)	0.959
Grade			
Junior	Ref	Ref	
Senior year	-0.041	(-0.404,0.322)	0.825
Fifth year	-0.087	(-0.533,0.358)	0.700
Knowledge	0.186	(0.101,0.271)	< 0.001
Attitude	0.593	(0.537,0.649)	< 0.001

### Mediation effect analysis

**Fig. 1:**
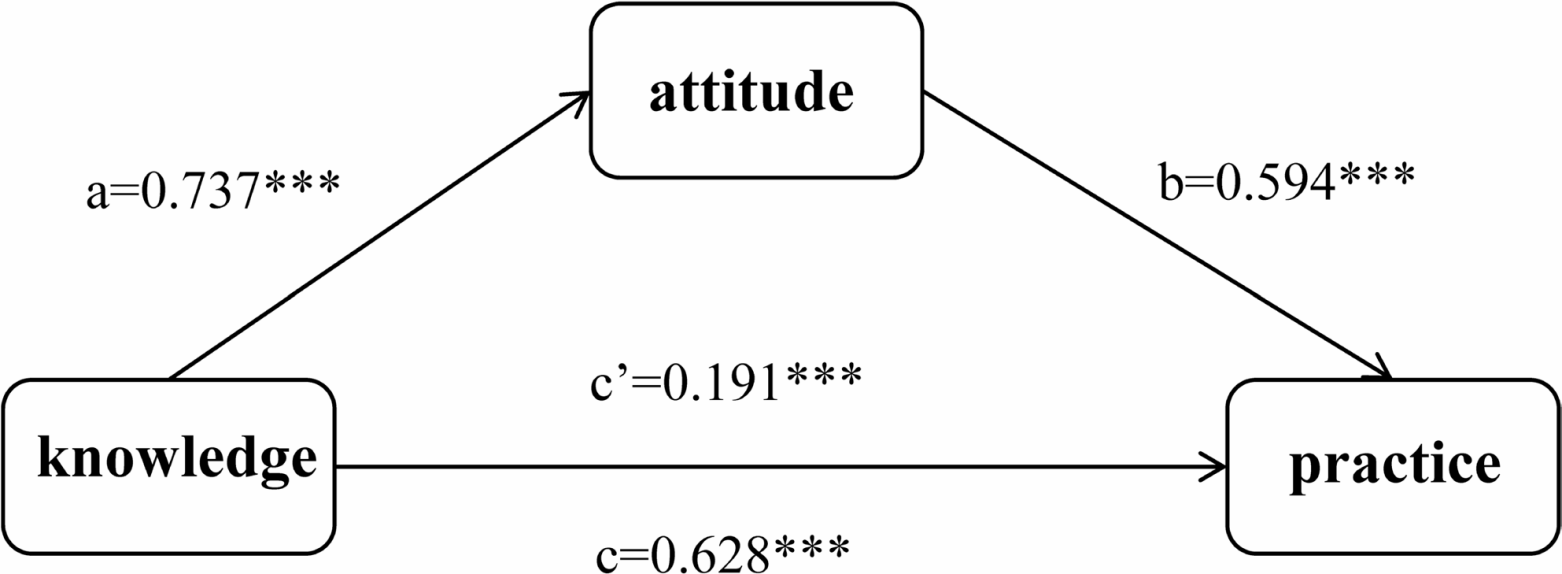
Mediating effect model. Note: ***P < 0.001, **P < 0.01, *P < 0.05

The total effect of knowledge on practice was significant (β = 0.628, 95% CI [0.523, 0.733], P < 0.001). After introducing attitude as a mediating variable, the direct effect of knowledge on practice remained significant (β = 0.191, 95% CI [0.114, 0.268], P < 0.001). Indirect effect analysis indicated that the pathway through attitude was also significant (indirect effect = 0.437, 95% Boot CI [0.348, 0.548]) (Table 9). Path analysis further showed that knowledge significantly and positively predicted attitude (path a = 0.737, P < 0.001), and attitude significantly and positively predicted practice (path b = 0.594, P < 0.001). The total effect (path c = 0.628, P < 0.001) and the direct effect (path c’ = 0.191, *P* < 0.001) are illustrated in Fig. 1. The indirect effect accounts for 69.59% of the total effect. The model showed good fit: knowledge and attitude together explained 73.51% of the variance in practice (R²=0.735, F = 406.47, *P* < 0.001).

**Table 9:** Mediating effect analysis

Path/Effect	Coefficient	SE	95%CI	Bootstrap 95%CI	*P*
Total effect (knowledge→practice)	0.628	0.053	[0.523,0.733]	-	< 0.001
Direct effect (knowledge→practice)	0.191	0.039	[0.114,0.268]	-	< 0.001
Indirect effect	0.437	0.051		[0.348,0.548]	-
Knowledge→attitude (path a)	0.737	0.070	[0.599,0.876]	-	< 0.001
Attitude→practice (path b)	0.594	0.028	[0.539,0.648]	-	< 0.001

## Discussion

Chinese medical students generally demonstrate adequate knowledge, positive attitudes, and modest level of practices toward virtual simulation. These findings underscore the potential of virtual simulation as a transformative tool in medical education. Future efforts should focus on improving accessibility, integrating blended learning approaches, and conducting longitudinal research to evaluate long-term educational outcomes.

The findings of this study align with and extend existing research on virtual simulation in medical education. The high level of knowledge and positive attitudes observed among students corroborate previous studies highlighting the efficacy of virtual simulation in enhancing learning outcomes [1, 4]. For instance, de Toro Santos et al. (2024) emphasized the utility of virtual reality in clinical skill mastery, particularly in procedural training [1]. Similarly, Zhang and Liu’s meta-analysis (2022) underscored the medium-to-high impact of augmented reality and virtual simulation technologies in education, supporting our results [4].

The mediating role of attitudes in the KAP model is consistent with Alzghoul’s (2015) framework, which posits that attitudes significantly influence the translation of knowledge into practice [8]. This study further validates the KAP model’s applicability in the context of virtual simulation, demonstrating that students with greater knowledge and more positive attitudes are more likely to engage in virtual training. Similar patterns have been reported in international studies. Research conducted in Western educational settings has shown that medical students generally exhibit positive attitudes toward simulation-based learning and that favorable perceptions are associated with higher engagement and skill acquisition [2, 7]. Studies from Middle Eastern contexts using the KAP framework have also demonstrated that attitude plays a critical role in translating knowledge into practice-related behaviors [11]. These findings suggest that the observed relationships among knowledge, attitude, and practice in virtual simulation training may reflect a broadly shared learning mechanism among medical students across different cultural and educational systems, rather than being limited to the Chinese context. From a practical perspective, the strong mediating effect of attitude suggests that educational institutions should not only focus on technical instruction and operational training, but also emphasize the perceived educational value and learning benefits of virtual simulation. Strategies such as showcasing successful learning outcomes, integrating simulation into assessment systems, and highlighting its relevance to clinical competence may help strengthen positive attitudes and, in turn, more effectively translate knowledge into sustained learning practice.

However, our results also highlight disparities in the adoption of virtual simulation across different medical specialties. Radiology and Anesthesiology students exhibited higher knowledge scores compared to Clinical Medicine students, possibly due to the technical nature of their disciplines. In particular, the higher knowledge levels observed among Radiology students can be partially ascribed to the inherently digital and image-based nature of radiology training, which routinely involves advanced imaging systems, digital workstations, and computer-assisted diagnostic tools. Such learning environments are more likely to enhance students’ familiarity with technology-enhanced instruction and facilitate greater acceptance and understanding of virtual simulation platforms compared with more traditional bedside-oriented clinical training. This finding echoes Li et al.’s (2016) observation that virtual simulation’s effectiveness varies by subject, necessitating tailored implementation strategies [6]. The preference for virtual simulation during clinical specialty courses (76.4%) aligns with You et al. (2020) recommendation for its use in advanced training phases [2]. Yet, challenges such as the rigidity of virtual scenarios [11] and the need for blended learning approaches [15] were noted, reflecting broader concerns in the literature. Wang et al.. (2024) advocated for a “virtual-actual combination” to balance immersive technology with hands-on experience, a suggestion supported by our participants’ feedback [11].

The findings of this study indicate that the majority of medical students have a comprehensive understanding of virtual simulation technology and acknowledge its significant value in medical education. Over half of the medical students hold the view that virtual simulation teaching surpasses traditional skills training in terms of advantages. It can enhance the realism and immersive experience of the learning process, allowing students to conduct independent explorations repeatedly and fully develop their practical abilities. Virtual simulation teaching also optimizes the efficiency of teaching resource utilization. As noted by Li et al.. (2014) [20], it reduces the costs associated with experimental training and minimizes resource consumption. Additionally, it promotes the efficient allocation and sharing of teaching resources, ensuring that high-quality educational resources can benefit a larger number of students. Moreover, virtual simulation teaching software can be customized according to students’ learning progress, interests, and ability levels, tailored for personalized learning paths, offering teaching content with varying degrees of difficulty, to meet the diverse learning needs of students from different backgrounds. Furthermore, the online virtual simulation platform provides students with the convenience of learning anytime and anywhere, and the advantage is vividly illustrated by the platform’s data. On China’s largest virtual simulation experimental teaching course sharing platform, a large number of registered students from diverse regions and institutions have gathered. According to the Higher Education Press Experimental Space Operation Working Group (2020), the average number of experiments conducted by student users on this platform is 8 times.

In the multivariate linear regression analysis, practice was significantly associated with knowledge (β = 0.186, *P* < 0.001) and attitude (β = 0.593, *P* < 0.001), whereas no significant associations were observed between demographic variables and practice. This suggests that medical students with higher levels of knowledge and more positive attitudes tend to exhibit stronger engagement in virtual simulation learning. As demonstrated by Alzghoul (2015), individuals with higher knowledge levels and more favorable attitudes are more likely to translate cognition into effective practice within the KAP framework [11].

However, as virtual simulation technology is applied, certain drawbacks have gradually emerged. Its process is relatively rigid and often involves mechanical repetition. Relying solely on virtual simulation teaching may lead to a subpar learning experience for students [21]. In contrast, the blended teaching mode based on virtual simulation technology effectively combines the advantages of virtual simulation with the essential elements of classroom teaching, resulting in a favorable teaching outcome [22].

As an innovative driving force in the education field, virtual simulation teaching harbors immense development potential and broad application prospects. Virtual simulation teaching is increasingly regarded as a pivotal component in modern higher education, thanks to its immersive, repeatable and scalable features, which allow for enhanced student engagement, skill acquisition, and cost-efficient learning environments [23]. The profound integration of virtual simulation instruction with technologies like artificial intelligence, augmented reality, and virtual reality unlocks boundless possibilities for teaching innovation [24]. The implementation of intelligent tutoring, personalized recommendation of teaching content, and intelligent evaluation and feedback mechanisms render the teaching process more precise and efficient [25]. This, in turn, enables it to better satisfy the individualized needs of students.

Future research should prioritize multi-center studies to validate these findings across diverse populations and institutions. Longitudinal designs could track how KAP evolves over time and assess the long-term impact of virtual simulation on clinical performance. Additionally, exploring the effectiveness of blended learning models that combine virtual and hands-on training may optimize skill acquisition. Technological advancements, such as AI-driven personalization and augmented reality, should be leveraged to address current limitations like scenario rigidity. Collaborative efforts between educators, researchers, and developers will be essential to refine virtual simulation tools and ensure equitable access. By pursuing these directions, virtual simulation can realize its full potential as a transformative force in medical education, preparing students to meet the demands of modern healthcare with confidence and competence [16, 17].

### Limitations

This study has several limitations. First, the use of a self-designed questionnaire may introduce measurement bias, despite its validation through pre-testing. Second, the sample was drawn from a single institution, limiting the generalizability of the findings. However, the relatively large sample size and high response rate may partially strengthen the internal validity of the study and reduce the risk of non-response bias. A nationally representative sample would provide a more comprehensive understanding of KAP across diverse educational and regional contexts. Third, the cross-sectional design precludes causal inferences; longitudinal studies are needed to assess how KAP evolves over time. Finally, the study did not evaluate the long-term impact of virtual simulation on clinical performance, an area warranting further investigation.

## Conclusion

Chinese medical students enrolled at Anhui Medical University generally exhibited adequate knowledge and positive attitudes toward virtual simulation, while actual engagement in virtual simulation training remained limited. Students’ understanding and application of virtual simulation can be enhanced through intensified publicity and training, thus promoting their active engagement in virtual simulation learning. Furthermore, educational institutions have the opportunity to design virtual simulation programs tailored specifically for Clinical Medicine students or introduce clinical skills training courses grounded in virtual simulation technology. Such initiatives can more effectively facilitate the development of students’ clinical skills. It is advisable to primarily target students majoring in Clinical Medicine for this training, while also including other students who are required to take the licensing examination. Virtual simulation training can be implemented during students’ learning of clinical medicine courses or at their internship stage, providing them with practical and immersive learning experiences.

## Supplementary Information

Supplementary Material 1.

## Data Availability

The authors confirm that the data supporting the findings of this study are available within the article.

## Abbreviations

KAP: Knowledge, attitudes, and practices
SD: Standard deviation
