# Supplementary material for: Knowledge, attitudes, and practices of medical students at a Chinese university toward virtual simulation experimental training: a cross-sectional survey of self-perceived experience
Source: BMC Med Educ. 2026 Feb 12;26:447. doi: 10.1186/s12909-026-08803-w (PMC13001291; doi:10.1186/s12909-026-08803-w)
Supplement: Supplementary file 1 — Supplementary Material 1. [file 12909_2026_8803_MOESM1_ESM.docx]

**Survey on Virtual Simulation Experimental Training**

Dear students,

Thank you for taking time to participate in this survey. The purpose of this survey is to understand the basic situation and development trends of virtual simulation experimental training. Please fill it out truthfully according to your actual situation. We will keep your answers confidential. Continuing to answer indicates that you have been informed and agreed.

Thank you for your support and cooperation!

Education Division,

The First Affiliated Hospital of Anhui Medical University

1. Name [Fill-in-the-blank questions]

_________________________________

2. Gender [Fill-in-the-blank questions]

| ○Male | ○Female |  |  |  |  |  |  |
| --- | --- | --- | --- | --- | --- | --- | --- |

3. School [Fill-in-the-blank question]

_________________________________

4. Major [Fill-in-the-blank question]

_________________________________

5. Grade [Fill-in-the-blank question]

_________________________________

6. Do you know about virtual simulation technology? [Multiple-choice question]

| Strongly Disagree | ○1 | ○2 | ○3 | ○4 | ○5 | Strongly Agree |
| --- | --- | --- | --- | --- | --- | --- |

7. Do you know about medical virtual simulation experimental training teaching? [Multiple-choice question]

| Strongly Disagree | ○1 | ○2 | ○3 | ○4 | ○5 | Strongly Agree |
| --- | --- | --- | --- | --- | --- | --- |

1. What is the way you learned about virtual simulation? [Multiple-select question]

□ Lectures or conferences

□ Internet (social media platforms, Moments, official WeChat accounts)

□ Textbooks, professional journals or related works

□ Course training

□ Recommendations from others

□ Never heard of it

9. Do you think virtual simulation experimental training is more advantageous than traditional skills training? [Multiple-choice question]

| Strongly Disagree | ○1 | ○2 | ○3 | ○4 | ○5 | Strongly Agree |
| --- | --- | --- | --- | --- | --- | --- |

10. What do you think are the advantages of virtual simulation technology? [Multiple-select question]

□ Clinical simulation and immersion

□ Human–computer interaction and timely feedback

□ Rich resources for independent learning

□ Safety for practical training

11. Are you willing to participate in virtual simulation clinical skills training? [Multiple-choice question]

| Strongly Disagree | ○1 | ○2 | ○3 | ○4 | ○5 | Strongly Agree |
| --- | --- | --- | --- | --- | --- | --- |

1. At which stage do you think it is necessary to conduct virtual simulation for clinical learning? [Multiple-select question]

□ Before learning clinical specialty courses

□ While learning clinical specialty courses

□ During clinical internship

□ Not interested

13. Do you hold a positive attitude towards virtual simulation learning’s ability to stimulate learning enthusiasm and improve independent learning efficiency? [Multiple-choice question]

| Strongly Disagree | ○1 | ○2 | ○3 | ○4 | ○5 | Strongly Agree |
| --- | --- | --- | --- | --- | --- | --- |

14. Do you think virtual simulation learning can achieve results comparable to real experiments and clinical practice? [Multiple-choice question]

| Strongly Disagree | ○1 | ○2 | ○3 | ○4 | ○5 | Strongly Agree |
| --- | --- | --- | --- | --- | --- | --- |

15. Are you willing to recommend virtual simulation experimental training to others? [Multiple-choice question]

| Strongly Disagree | ○1 | ○2 | ○3 | ○4 | ○5 | Strongly Agree |
| --- | --- | --- | --- | --- | --- | --- |

16. Have you ever participated in a virtual simulation experimental training course? [Multiple-choice question]

| ○Yes |
| --- |
| ○No |

17. Have your experimental or clinical skills been improved through virtual simulation learning? [Multiple-choice question]

| Strongly Disagree | ○1 | ○2 | ○3 | ○4 | ○5 | Strongly Agree |
| --- | --- | --- | --- | --- | --- | --- |

1. In what way will you engage in learning through virtual simulation training? [Multiple-select question]

□ Take courses at your own school

□ Use public e-learning platforms

□ Take courses offered by other schools

□ Other methods _________________

□ Not interested

19. Have you used virtual simulation courses to replace part of real experimental or clinical skills training? [Multiple-choice question]

| Strongly Disagree | ○1 | ○2 | ○3 | ○4 | ○5 | Strongly Agree |
| --- | --- | --- | --- | --- | --- | --- |

20. If virtual simulation experimental training is offered as a compulsory undergraduate course, will you participate? [Multiple-choice question]

| Strongly Disagree | ○1 | ○2 | ○3 | ○4 | ○5 | Strongly Agree |
| --- | --- | --- | --- | --- | --- | --- |

21. What learning problems do you hope virtual simulation training can solve? [Fill-in-the-blank question]

_________________________________
